# Effectiveness of a clinical pathway for acute stroke care in a district general hospital: an audit

**DOI:** 10.1186/1472-6963-6-16

**Published:** 2006-02-23

**Authors:** William J Taylor, Annie Wong, Richard J Siegert, Harry K McNaughton

**Affiliations:** 1 Rehabilitation Teaching & Research Unit, Wellington School of Medicine & Health Sciences, University of Otago, PO Box 7343, Wellington, New Zealand; 2 Medical Research Institute of New Zealand, PO Box 10055, Wellington, New Zealand

## Abstract

**Background:**

Organised stroke care saves lives and reduces disability. A clinical pathway might be a form of organised stroke care, but the evidence for the effectiveness of this model of care is limited.

**Methods:**

This study was a retrospective audit study of consecutive stroke admissions in the setting of an acute general medical unit in a district general hospital. The case-notes of patients admitted with stroke for a 6-month period before and after introduction of the pathway, were reviewed to determine data on length of stay, outcome, functional status, (Barthel Index, BI and Modified Rankin Scale, MRS), Oxfordshire Community Stroke Project (OCSP) sub-type, use of investigations, specific management issues and secondary prevention strategies. Logistic regression was used to adjust for differences in case-mix.

**Results:**

N = 77 (prior to the pathway) and 76 (following the pathway). The median (interquartile range, IQR) age was 78 years (67.75–84.25), 88% were European NZ and 37% were male. The median (IQR) BI at admission for the pre-pathway group was less than the post-pathway group: 6 (0–13.5) vs. 10 (4–15.5), p = 0.018 but other baseline variables were statistically similar. There were no significant differences between any of the outcome or process of care variables, except that echocardiograms were done less frequently after the pathway was introduced. A good outcome (MRS<4) was obtained in 66.2% prior to the pathway and 67.1% after the pathway. In-hospital mortality was 20.8% and 23.1%. However, using logistic regression to adjust for the differences in admission BI, it appeared that admission after the pathway was introduced had a significant negative effect on the probability of good outcome (OR 0.29, 95%CI 0.09-0.99).

**Conclusion:**

A clinical pathway for acute stroke management appeared to have no benefit for the outcome or processes of care and may even have been associated with worse outcomes. These data support the conclusions of a recent Cochrane review.

## Background

A number of randomised controlled trials and systematic reviews show that organised stroke care, mainly in the form of a stroke unit, reduces early mortality and improves disability for patients with stroke [1]. The implementation of this research into clinical practice has been slow. Only recently have most United Kingdom hospitals established stroke units, although only a minority of stroke patients appear to access them [2]. In New Zealand, very few hospitals have developed organised stroke care [3]. One of the barriers to developing a dedicated stroke unit may be the re-distribution of resources away from acute medical services or the investment of new resources.

An alternative way of delivering organised stroke care may be a clinical pathway. This require fewer resources, being mainly a tool for encouraging clinicians to implement evidence-based management decisions at key stages during a patient's hospitalisation. The assumption is that the components of a stroke unit could be mimicked by general medical care if information about these components were explicit and available at the salient point of patient care. Clinical pathways have been promoted in a number of medical conditions such as acute coronary syndromes [4], exacerbations of chronic obstructive airways disease [5], and peptic ulcer disease [6]. The evidence for the effectiveness of clinical pathways in stroke is mainly limited to non-randomised studies and is consequently not strong. A systematic review of 3 randomised and 7 non-randomised studies concluded that there is no evidence that clinical pathways improve mortality or dependency for patients with stroke [7]. Despite the poor evidence base, clinical pathways are still recommended as a means of promoting best practice in hospital care for people with stroke [8].

In April 2003, a clinical pathway was introduced in Hutt Hospital to promote best practice for patients with acute stroke admitted to the general medical service. Given the limited evidence for clinical pathways in stroke care, it was felt important to audit the outcomes and processes of care shortly after implementation of the pathway. The principal aim of this audit was to assess whether the outcomes at discharge were improved for patients with stroke, after the pathway was introduced compared to a cohort managed in hospital prior to the pathway. We also assessed whether there were changes in the use of investigations, management of specific problems in acute stroke and the use of secondary prevention treatment at discharge from hospital.

## Methods

The design of this audit was a retrospective case-note review of two groups of consecutive patients with stroke admitted to a district general hospital serving an urban population of 135,000 people. About 150 patients with stroke are admitted annually to the general medical service. Neurology and neuro-surgical services are provided at a regional centre about 25 minutes drive from Hutt Hospital. Inpatient rehabilitation is provided within an age-unrelated rehabilitation service on the hospital campus.

The stroke pathway consisted of a paper document inserted into each stroke admission, either in the emergency department or medical unit. It contained general advice about acute stroke management and then detailed daily activities for each of the first 5 days of the acute admission. The daily activities were listed under medical, nursing, therapist and discharge planning headings. Each item was to be ticked, initialled and dated as achieved by relevant staff on a daily basis. The content of the pathway was determined by literature review, consensus statements and regular meetings of a stroke pathway team over a 3-year period.

Cases were identified from International Codes for Diagnosis (ICD-10) discharge codes for the 6-month periods of June through December 2003 (pre-pathway group) and June through December 2004 (post-pathway group). In addition, after the pathway was introduced a logbook was kept that prospectively recorded patients with stroke admitted to the medical unit; additional cases were identified from this source. The case-notes were reviewed to confirm that the primary reason for admission was stroke according to the World Health Organisation definition [9].

The following data were abstracted from the case-notes of confirmed stroke patients: length of stay on medical and rehabilitation services, demographics, neurological impairments necessary to classify the stroke according to the Oxfordshire Community Stroke Project (OCSP) [10], Charlson Comorbidity Index [11], Barthel Index [12] at admission and discharge, estimated Modified Rankin Scale [13] prior to admission and at discharge, discharge disposition, presence and management of specific issues (fever, hyperglycemia, hypertension, prophylaxis for thromboembolism, aspirin given within 48 hours of admission), use of investigations (computed tomography, magnetic resonance imaging, echocardiography, carotid doppler ultrasound, fasting lipid profile, acute phase marker [erythrocyte sedimentation rate or C-reactive protein]), and use of secondary prevention treatment at discharge (blood pressure lowering treatment, smoking cessation programme, optimisation of diabetic control, cholesterol lowering treatment, anti-platelet treatment, anticoagulation for atrial fibrillation).

The Barthel Index (BI) is a 10-item scale of independence in mobility and self-care activities, with scores ranging from 0 (complete dependence) to 20 (complete independence). Scores of 0 to 10 typically indicate severe functional disability. Although the BI was not routinely administered at admission and discharge during either period, it was possible to retrospectively score the items by reference to nursing, occupational therapy and physiotherapy notes within the case-record. The Modified Rankin Scale (MRS) is a simple 6-level categorisation of functional independence: 0 indicates no symptoms, 1 indicates symptoms but no disability, 2 indicates slight disability but independent, 3 indicates moderate disability but can walk independently, 4 indicates moderately severe disability, 5 indicates severe disability. In-hospital functional status at admission was measured with the BI rather than the MRS since the BI is more informative and the MRS is more frequently employed as a community-based outcome, rather than an adequately scaled functional index for hospital inpatients [14].

A good outcome at discharge from hospital was defined in two ways: discharge to home, nearly independent survival (MRS<4) and independent survival (MRS<3). A MRS<4 has not previously been used to define a good outcome in acute stroke trials, but was chosen here to capture modest improvements in patients severely affected by stroke.

Non-parametric statistical tests were used to evaluate differences between the two cohorts (Mann-Whitney U for continuous or ordinal data, Chi-square for categorical data). To adjust for differences in case-mix [15], the influence of being admitted in the post-pathway group upon outcome was assessed using logistic regression models incorporating age, gender, comorbidity index, admission BI, whether transferred to rehabilitation and OCSP classification. SPSS version11.5 was used for all analyses.

## Results

**Figure 1:**
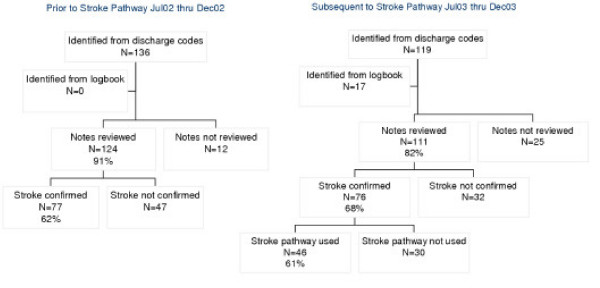
Case identification flow-chart.

In the pre-pathway period, 136 patients had discharge codes that indicated stroke. The case-records of 91% of these were examined and 77 (62%) were confirmed to have a primary admission reason of stroke (Figure 1). In the post-pathway period, 119 patients had discharge codes that indicated stroke and an additional 17 patients were identified from the stroke logbook. Of these, 82% of the case-records were examined and 76 (68%) were confirmed to have a primary admission of stroke. There was documented evidence that the stroke pathway was used in 61% of these patients.

The two cohorts were similar in terms of age, OCSP classification, gender and ethnicity but the pre-pathway group had worse functional status at admission (Table 1).

**Figure 2:**
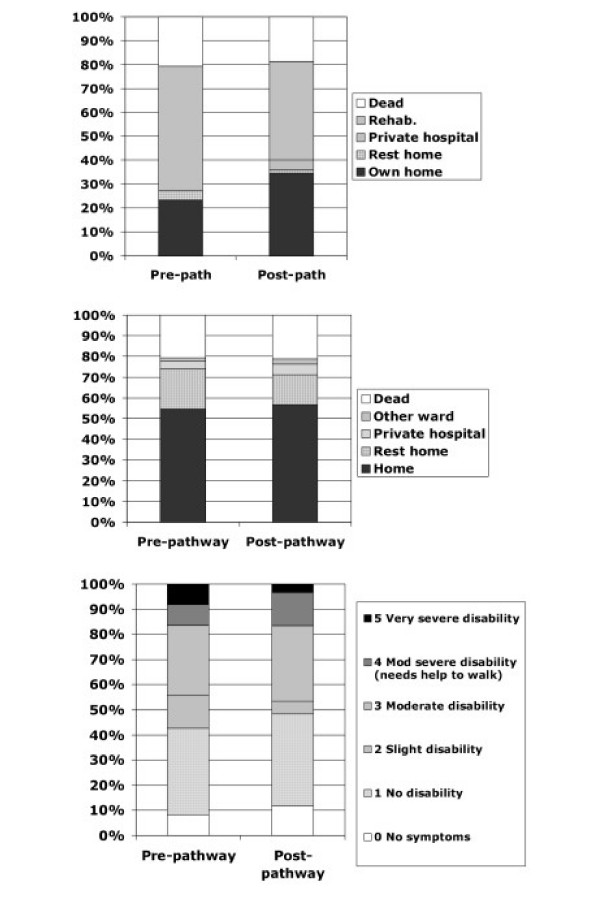
Outcomes at hospital discharge. Top: Discharge location from medical unit. Centre: Discharge location from hospital. Bottom: Modified Rankin Scale of survivors at discharge from hospital.

Length of stay (LOS) on medical or rehabilitation units was not different between the two cohorts (Table 2). Although there was a trend to shorter total hospital LOS (a difference in median LOS of 5.5 days following the introduction of the pathway due to fewer patients being transferred to rehabilitation) (Figure 2), this apparent difference was not statistically significant.

Discharge disposition from hospital was very similar in the two cohorts but there was a trend towards fewer patients transferred to inpatient rehabilitation following introduction of the pathway and more patients discharged directly home from the acute medical unit (Figure 2). Similarly there were no differences in the proportion of survivors at discharge classified by MRS.

Since there were differences in admission BI between the two cohorts, we adjusted for this using logistic regression. Using logistic regression to model the logit probability of a good outcome (defined as MRS<4 at discharge) with age, admission BI, transfer to inpatient rehabilitation, admission during the pathway period, comorbidity score, and OCSP subtype as independent variables, we found that admission during the pathway period had an independent adverse effect on outcome (Table 3). This effect remained if the definition of a good outcome was MRS<3 (model not shown), but the independent effect of transfer to rehabilitation was not significant in this second model. A similar analysis was performed excluding patients for whom there was no evidence of pathway use in the post-pathway period, and very similar findings were obtained (data not shown).

Table 4 lists the use of investigations, management of specific issues and secondary risk factor management. There were significantly fewer echocardiograms performed during the hospital stay after the pathway was introduced but no other significant differences in the use of investigations were observed. Fewer than half of patients had fasting lipids and glucose measured during the hospital stay, which is unsatisfactory. There were no significant differences in the early use of aspirin, paracetamol for fever, treatment of acute hyperglycemia, acute blood pressure management or the use of DVT prophylaxis. Neither were there differences in the proportions of patients having appropriate secondary prevention treatment at discharge. Under-treatment with warfarin for atrial fibrillation seems likely in both cohorts.

## Discussion

This observational retrospective audit does not provide evidence to support the use of a clinical pathway in an acute setting for stroke patients, when the objective of such a pathway is to improve discharge outcomes. There are other possible advantages to clinical pathways such as improved documentation, compliance with funding requirements and ease of audit, but this study did not address these issues. Clearly, the non-randomised design and retrospective design does not permit straightforward interpretation of the finding that admission in the post-pathway period was associated with an adverse outcome. It may be that other un-measured variables could account for this finding, such as changes in staffing levels, provision of residential care or differences in informal supports available to patients.

One limitation to the retrospective design is the likelihood that not all stroke admissions were identified. The inaccuracy of discharge coding is well known. However, it is likely that such a bias was present to the same extent in both cohorts since we found that confirmed strokes accounted for a similar proportion of cases identified from discharge codes in each group.

Similarly, retrospective determination of the admission Barthel Index from case-notes is unlikely to be strictly accurate. However, the same method of determining BI was used for both pre- and post-pathway groups, so a systematic bias is unlikely. Also, the calculation of the Barthel Index was not blinded to whether the sample was before or after the introduction of the pathway. We cannot exclude the possibility that this could have affected the scores and could conceivably led to the observed difference in mean Barthel Index scores at admission. However, we feel that this is unlikely since the trends in other data also suggested that strokes admitted after introduction of the pathway were milder – shorter total hospital length of stay and fewer strokes transferred to rehabilitation.

It is also possible that admission practices changed after the introduction of the pathway. Although there was no official policy change, the emphasis on hospital management for stroke probably meant that increased numbers of less severe strokes were admitted after the introduction of the pathway; this is strongly suggested by the better admission functional status and the increased proportion of patients discharged directly home from the acute medical unit. This difference in case-mix does confound the interpretation of outcomes between the two cohorts, but statistical adjustment suggests a worse outcome for strokes admitted after the pathway was introduced.

A Cochrane systematic review of in-hospital care pathways for stroke showed that evidence from mainly non-randomised studies suggests that stroke pathways may reduce urinary tract infection and re-admission rates and increase rates of having a CT brain study or carotid duplex study [7]. However, there was no evidence that in-hospital pathways reduced length of stay or improved outcome at discharge (death, dependency or destination). Furthermore, evidence from randomised trials suggested that quality of life and patient satisfaction were worse in patients on a stroke pathway.

If it is true that stroke pathways fail to improve outcome at discharge and may even be associated with an adverse effect upon outcome, it is interesting to speculate why this might be so. It may be that the complexity of acute stroke care cannot be adequately described within a clinical pathway and that simple guidelines cannot hope to replace experienced, knowledgeable staff who can think flexibly. It may be that pathways channel clinical staff into routines that may not be best practice for individual patients.

We have previously demonstrated an ambiguous relationship between process of care and outcomes for stroke patients [16]. However, it has been suggested that relatively simple measures might be able to reduce unnecessary catheter use and prevent aspiration [17] without implementing a full clinical pathway. Certainly, a clinical pathway is not without some opportunity cost: multidisciplinary staff time in meetings, researching the literature and writing the pathway, staff training (which needs to be regularly updated), and in promoting compliance with the pathway.

## Conclusion

It seems likely that the as-yet-unidentified components of stroke care within comprehensive stroke units that account for the effectiveness of such units, are not contained within the clinical pathway approach. This audit strengthens the argument for stroke unit care to be the service delivery model of choice for patients admitted with stroke.

## List of abbreviations

BI Barthel Index

MRS Modified Rankin Scale

ICD-10 International Classification of Disease, 10^th ^edition

OCSP Oxfordshire Community Stroke Project

SPSS Statistics Package for the Social Sciences

LOS Length of stay

## Competing interests

The author(s) declare that they have no competing interests.

## Authors' contributions

WJT conceived of the study, supervised data collection, performed the data analysis and drafted the manuscript. AW collected the data, performed initial data analysis and helped to draft the manuscript. RJS helped design the study and helped to draft the manuscript. HKM helped design the study and helped to draft the manuscript. All authors read and approved the final manuscript.

## Pre-publication history

The pre-publication history for this paper can be accessed here:



## Figures and Tables

**Table 1:** Baseline characteristics

		Pre-path	Post-path	p-value
Age (median, IQR)		78(70–86)	77(65–83)	NS
Gender (Male N, %)		34(44.2)	33(43.4)	NS
Ethnicity (European N, %)		64(83.1)	69(90.8)	NS
Barthel Index admission (median, IQR)		6(0–13.5)	9.5(4–15)	0.025
OCSP subtype (N, %)	TACS	5(6.5)	5(6.5)	NS
	PACS	40(51.9)	31(40.8)	
	POCS	5(6.5)	11(14.5)	
	LACS	20(26)	20(26.3)	
	Unclassified	7(9.1)	9(11.8)	

**Table 2:** Length of stay (LOS), median (IQR)

	Pre-path	Post-path	p-value
Medical ward (days)	6(3–9)	5.5(3–9)	NS
Rehabilitation ward (days)	21(13.25–30.75	21(15–31)	NS
Total hospital LOS	16(4–30)	10.5(4–26.5)	NS

**Table 3:** Logistic regression model for discharge MRS<4 (-2log likelihood 94.9, R^2 ^0.48, p < 0.001)

		B(SE)	OR(95%CI)	p-value
Comorbidity index		-0.28(0.24)	0.76(0.47–1.22)	0.25
Age		0.01(0.02)	1.01(0.96–1.06)	0.81
Admission BI		0.39(0.07)	1.48(1.29–1.70)	<0.001
OCSP class	TACS		1.00 (ref)	
	PACS	-0.11(0.88)	0.90(0.16–5.06)	0.90
	POCS	2.10(1.44)	8.16(0.48–138)	0.15
	LACS	-0.90(1.16)	0.41(0.04–3.90)	0.43
Post-pathway period		-1.23(0.63)	0.29(0.09–0.99)	0.05
Female gender		0.57(0.59)	1.78(0.56–5.63)	0.33
Transfer to Rehab.		1.27(0.56)	3.55(1.19–10.63)	0.02

**Table 4:** Medical management of stroke patient during hospital stay

		Before the introduction of pathway	After the introduction of pathway	p-value
Investigations during the admission	CT scan in 1st 48 hrs	96%	92%	0.293
	Carotid duplex ultrasound	26%	28%	0.817
	Echocardiogram	42%	26%	0.047
	ESR or CRP in 1st 48 hrs	57%	61%	0.143
	Fasting blood sugar and lipids	32%	44%	0.143
	MRI	10%	5%	0.238

Specific medical issues	Aspirin given in first 48 hours to patients without contraindications	26/55 (46%)	40/66 (61%)	0.117
	Paracetamol given to those with fever > 38°C in first 48 hours	3/4 (75%)	5/5 (100%)	0.574
	Acute blood pressure management consistent with guideline	63.6%	71.1%	0.328
	Prophylaxis for deep vein thrombosis (except aspirin)	20.8%	14.5%	0.306
	Hypoglycemic agents given to those with blood sugar >15 mmol/l in first 48 hours	1/2 (50%)	2/2 (100%)	0.312

Risk factor treatment at discharge	Anti-hypertensive treatment if sBP>140 or dBP>90 at any time during admission	44/50 (88%)	51/58 (88%)	0.242
	Warfarin if ECG shows atrial fibrillation	6/18 (33%)	4/10 (40%)	0.264
	Statin if total cholesterol > 3.5	42/55 (76%)	48/61 (79%)	0.341
	Cessation programme for current smokers	5/10 (50%)	8/10 (80%)	0.638
	Optimisation of glycemic control if diabetes present	8/12 (67%)	8/9 (100%)	0.410
	US carotid arteries requested or performed if anterior circulation infarct	19/60 (32%)	24/59 (41%)	0.610
